# Taxonomic and Functional Ant Diversity Along tropical, Subtropical, and Subalpine Elevational Transects in Southwest China

**DOI:** 10.3390/insects10050128

**Published:** 2019-05-03

**Authors:** Alyssa M. Fontanilla, Akihiro Nakamura, Zhenghui Xu, Min Cao, Roger L. Kitching, Yong Tang, Chris J. Burwell

**Affiliations:** 1 CAS Key Laboratory of Tropical Forest Ecology, Xishuangbanna Tropical Botanical Garden, Chinese Academy of Sciences, Menglun, Mengla, Xishuangbanna, Yunnan 666303 China; alyssa@xtbg.ac.cn (A.M.F.); caom@xtbg.ac.cn (M.C.); ytangnz@gmail.com (Y.T.); 2 University of Chinese Academy of Sciences, Beijing 100049, China; 3 Key Laboratory of Forest Disaster Warning and Control in Yunnan Province, College of Biodiversity Conservation and Utilization, Southwest Forestry University, Kunming, Yunnan 650224, China; xuzhenghui1962@163.com; 4 Environmental Futures Research Institute and Griffith School of Environment and Science, Griffith University, Nathan, QLD 4111, Australia; r.kitching@griffith.edu.au (R.L.K.); chris.burwell@qm.qld.gov.au (C.J.B.); 5 Biodiversity Program, Queensland Museum, South Brisbane, QLD 4101, Australia

**Keywords:** ants, elevation, diversity gradient, functional diversity, community assembly, China

## Abstract

Although elevational gradients of biodiversity have long been the topic of scientific research, information on patterns of, and processes that shape insect community structure across elevation is still lacking. Addressing this gap requires the use of both taxonomic and functional approaches when studying diversity across elevational gradients. In this study, we examined taxonomic and functional alpha and beta diversity of ant assemblages sampled along tropical, subtropical, and subalpine elevational transects in Yunnan Province, southwest China. Species richness was used to quantify taxonomic alpha diversity, and two indices (FD and FRic) were calculated using morphological measurements to quantify functional alpha diversity. Taxonomic and functional beta diversity were partitioned into their turnover- and nestedness-resultant components. Though temperature and functional alpha diversity decreased linearly with increasing elevation, taxonomic alpha diversity showed a significant logarithmic decrease, with few species present at elevations greater than 3000 m a.s.l. The turnover-resultant component of taxonomic beta diversity increased with increasing elevational distance, while the nestedness-resultant component of functional beta diversity increased with increasing elevational distance in the subtropical transect. The observed patterns of taxonomic and functional diversity reflected ants’ thermophilic nature, implying functional adaptations (i.e., nested functional diversity) at higher elevations where environmental conditions were unfavorable.

## 1. Introduction

The variation of species diversity with latitude and elevation has been the subject of numerous studies for over three centuries, having been mentioned in the scientific literature as early as the late 1700s [1] and discussed at the start of the 19th century in more detail by Willdenow [2] and, perhaps most notably, by Humboldt [3]. Species diversity is generally highest in the tropics and decreases with increasing latitude (a pattern commonly referred to as the “latitudinal diversity gradient” or LDG; [4]), while elevational gradients tend to show more varied patterns among different taxa (e.g., [5,6,7]). The two main ecological hypotheses developed to explain the LDG focus either on net primary productivity, which is highest in the tropics and decreases with increasing latitude, or niche relationships, which are represented by adaptations of species to biotic interactions and abiotic conditions in their habitats [8]. However, productivity appears to have a smaller contribution to the LDG, and the tendency of tropical regions to harbor higher numbers of rare and/or specialized species suggests that niche relationships and the effects of abiotic factors such as temperature on species diversity are more significant [8]. Various factors such as productivity, climatic conditions, area, geometric constraints, and source-sink dynamics have been tested as possible mechanisms driving elevational diversity gradients [9], but temperature, which decreases with increasing elevation, has been shown to be one of the best predictors of species richness in elevational diversity gradient studies involving different taxa and biogeographical areas (e.g., [10,11]).

Since taxonomic diversity is traditionally quantified in terms of species richness and can therefore be limited by the species concept, combining taxonomic and functional diversity provides more information on the patterns of biodiversity and the ecological processes behind them. Unlike taxonomic diversity, functional diversity is not concerned with systematic relationships among species, and quantifies biodiversity within a given community based on the interspecific variation of traits related to ecosystem functioning [12].

Functional diversity can also indicate which assembly rules are at work in a given community [12]. According to the trait dispersion model [13], community structure is primarily determined by three factors: environmental filtering, competition, and stochastic processes. Environmental filtering occurs when harsh environmental conditions allow only certain phenotypes to survive and proliferate in a given habitat. As a result, functional traits become clustered, causing the observed functional diversity to be lower than expected by chance. If biotic processes such as competition dominate, species with similar phenotypes are unable to coexist, causing an over-dispersion of functional traits and higher functional diversity than expected. Stochastic assembly, however, occurs when the structure of a community is not due to environmental filtering or competition and is not significantly different from what would be expected by chance.

Changes in species composition (beta diversity) can be attributed to species turnover (i.e., communities differing in composition due to species unique to each community) or nestedness (i.e., communities differing in composition due to one community having a subset of species in another community). Adding a functional approach to examining turnover and nestedness is necessary to better understand community assembly processes along environmental gradients [14], including elevation [15]. As opposed to species turnover, functional turnover is reflected by a high number of functional strategies unique to each community [14]. Similarly, functional nestedness occurs when the functional strategies found in a given community represent a subset of a community with higher functional diversity.

The importance of using both taxonomic and functional approaches in biodiversity studies along elevational gradients is further underscored by the tendency of taxonomic and functional diversity to show contrasting results. In a study on butterfly communities along two elevational transects in Greece, taxonomic beta diversity was explained mostly by species turnover while functional beta diversity was due to nestedness [16]. Additionally, while termite species richness in a tropical mountain in Brazil decreased with increasing elevation, functional alpha diversity did not show any significant pattern [17]. Due to the limited number of studies looking at both facets of biodiversity across elevations, however, we do not know how common these contrasting patterns are and why they occur.

Ants are ideal study organisms to address questions about species diversity and community ecology, not only because of their great ecological dominance, for example as predators, herbivores, seed-dispersers, and nutrient transporters, but also because of their high biomass and low mobility which make them easy to sample in large numbers. Species diversity of ants is expected to be highest in warmer climates (and consequently, in lower elevations and latitudes) as ants are generally thermophilic [18]. A recent study found that clades of ants in tropical areas were older than those found in higher latitudes, supporting the tropical conservatism hypothesis which states that the climate in non-tropical regions has been cold only relatively recently. Consequently, non-tropical regions have not had time to acquire species diversity comparable to that of tropical regions which have been warmer for a much longer period of evolutionary time [19]. In contrast to the relatively consistent latitudinal diversity gradient [19,20] (but see [21]), global ant elevational diversity patterns appear to be mostly hump-shaped with mid-elevation peaks in species richness [22]. Although there are many studies addressing changes in the taxonomic diversity of ants along gradients of elevation, there are few which investigate changes in functional ant diversity. Both taxonomic and functional ant diversity decreased with temperature in high elevations along an elevational transect in Switzerland [23]. Bishop et al. [15] conducted a study of ant assemblages along an elevational transect in South Africa, where they found that both total taxonomic and functional beta diversity increased with elevational distance. Taxonomic beta diversity was explained mostly by turnover, with little contribution by nestedness. Changes in functional beta diversity were explained by both turnover and nestedness which showed contrasting patterns with respect to elevational distance. Functional nestedness initially decreased with elevational distance at short distances then increased when elevational distance was over 1500 m, whereas functional turnover increased with elevational distance then decreased after peaking at intermediate distances. The study by Bishop et al. [15] was, however, conducted within a grassland biome using only pitfall trapping. To our knowledge, taxonomic and functional beta diversity of ants have not been comprehensively studied within forested habitats. More research is therefore needed to address our current lack of knowledge on the processes that shape ant assemblages in mountain systems.

For this study, we collected ants from three elevational transects in varying climatic regions (tropical, subtropical, and subalpine) within Yunnan Province. We then examined how taxonomic and functional alpha diversity changed with elevation, whether species composition varied between elevational bands within the same transect, whether taxonomic and functional beta diversity were driven by either turnover or nestedness, and if ant community structure was shaped by either stochastic or deterministic processes. We hypothesized that (1) both taxonomic and functional ant diversity are negatively correlated with elevation due to ants’ general preference for warmer climates; (2) taxonomic and functional beta diversity increase with increasing elevational distance; (3) taxonomic beta diversity is due to species turnover while functional beta diversity is due to both turnover and nestedness; and (4) environmental filtering determines community structure in all three transects, causing observed functional diversity to be lower than expected by chance.

## 2. Materials and Methods

### 2.1. Study Site

**Figure A1:**
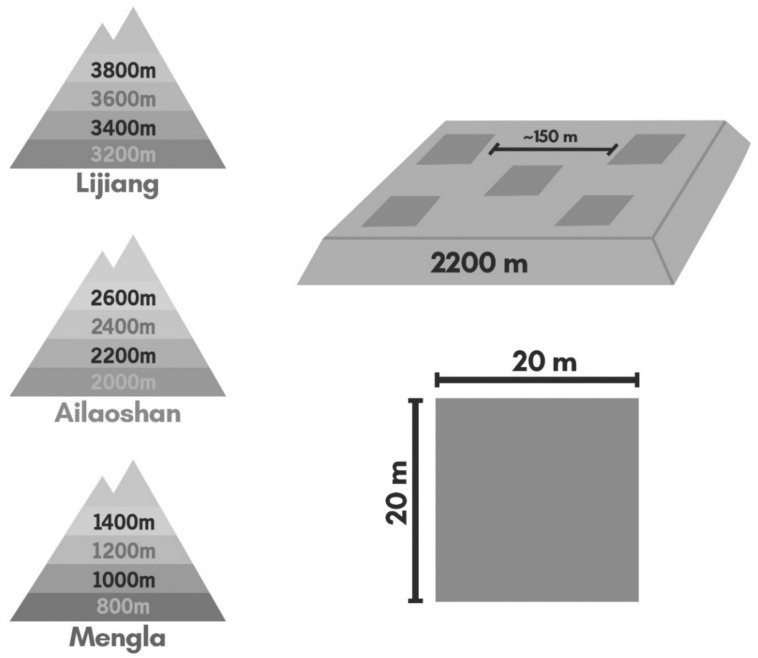
Schematic diagram of our study design, showing the elevational bands of the three transects (viz. Lijiang, Ailaoshan and Mengla; left), the distances between study plots within each elevational band, and the dimensions of each plot (bottom right). Distances are not to scale, and the precise spatial arrangement of study plots varied across elevations and transects depending on the available habitat.

Three elevational transects were established in Yunnan Province, southwest China, approximately 300 km apart along a north-south bearing: tropical Mengla (21.6°N, 101.5°E), subtropical Ailaoshan (24.2°N, 101.2°E), and subalpine Lijiang (27.1°N, 100.2°E) (see [24] for detailed site descriptions). Yunnan Province covers a large latitudinal and elevational range (21.2–29.2°N, 76.4–6740 m above sea level or a.s.l.) and is located between three geographic areas with distinct bioclimatic conditions: the Tibetan Plateau, the tropical southern Asia and Indo-China monsoon region, and the eastern Asia monsoon region [25]. Two biodiversity “hotspots”, South-Central China and Indo-Burma, also cover large parts of Yunnan [26]. The three transects were established as part of the Queensland-Chinese Academy of Sciences Biotechnology Project [24], which aimed to investigate the elevational distribution of forest biodiversity. Each transect was divided into four elevational bands separated by 200 m intervals, starting at 800 m a.s.l. in Mengla, 2000 m a.s.l. in Ailaoshan, and 3200 m a.s.l. in Lijiang (Figure A1 in Appendix A). At each elevational band, we established five 20 m × 20 m sampling plots spaced at least 150 m from each other. Plots were situated away from areas with visible disturbances, such as canopy gaps.

### 2.2. Field Sampling

Sampling was conducted on 1–20 July 2011 in Ailaoshan, 5–24 July 2012 in Mengla, and 9–22 August 2012 in Lijiang. Within each sampling plot, five sampling methods were employed following the protocol in Burwell and Nakamura [27]. These methods described below complement each other as they target different micro-habitats (e.g., leaf litter, soil surface, tree trunks) and activity times (diurnal and nocturnal ants).

- Litter extraction—Two sets of 1 m^2^ of leaf litter were collected at each plot. Leaf litter samples were taken just outside and on opposite sides of the plot to minimize disturbances within the plot, and to increase spatial coverage. Each 1 m^2^ sample was taken from four 0.25 m^2^ quadrats (50 cm × 50 cm) at least 5 m away from each other. Care was taken not to collect thick rain-washed deposits of litter and soil. From each quadrat, all litter and loose surface soil were collected by hand, sieved with a litter sifter, and processed using a Tullgren funnel for 24 to 36 hours, depending on the litter’s water content. Two sets of samples from each plot were pooled before analysis.
- Bark spray—We selected two sets of five trees greater than 30 cm dbh located outside and on opposite sides of the plot. We specifically targeted trees encrusted with vines, epiphytes, or moss to increase the number of ant species and individuals. We placed a rectangular sheet of nylon at the base of each tree and sprayed pyrethroid insecticide from the base to approximately 3 m up the trunk. Catches fallen into the nylon sheets were collected at least 15 minutes after spraying. As with the litter extraction method, two sets of samples from each plot were pooled before data analysis.
- Malaise trap—A Townes Malaise trap was set just outside each plot and operated for 10 days. Although this trapping method targets flying insects such as flies and wasps, wingless, primarily arboreal, ants were often collected.
- Pitfall traps—A total of 10 × 120 mL pitfall traps, each with an internal diameter of 44 mm and filled with 95% ethanol, were placed in each plot and left open for ten days. The 10 traps were installed along a diagonal line of the plot, approximately 2.5 m away from each other. A black plastic plate of 15 cm × 15 cm was suspended 4–5 cm above the pitfall traps to intercept rainfall. Samples from each plot were also pooled before data analysis.
- Hand collection—At each plot, hand collections were performed once within the 50 m radius of the plot for one hour during the day (09:00–17:00 h). Foraging ants and ants in nests were searched and collected by hand from the ground, foliage, and logs by CJB.

All samples were stored in 95% ethanol until ants were extracted. All worker ants were counted and identified to genus and then species or morphospecies by CJB and ZX. Reproductives were not included in this study. Ant specimens that represented different species or morphospecies at each elevational band were pinned. The majority of the specimen collection is currently stored in Southwest Forestry University, and a few specimens are stored in Xishuangbanna Tropical Botanical Garden.

**Table 1:** Morphological traits used for functional diversity analyses.

Trait	Justification	References
Weber’s length (WL)	Commonly used as a proxy for ant body size, which reflects the amount and type of resource exploited. May also reflect ability to nest in vegetation.	[15,28,29]
Relative eye length (RelEL)	While eyes are used by ants for navigation and recognition of predator and prey, eye size may also be indicative of when (i.e., diurnal or nocturnal) and where ants forage, as well as their dietary preference (predators tend to have smaller eyes than omnivores).	[29,30,31]
Relative scape length (RelSL)	May determine ants’ effective range of sensitivity to chemosensory signals	[31]
Relative mandible length (RelML)	Indicator of diet type, with predatory ants having longer mandibles	[31,32]
Relative hindleg length (RelHL)	Related to locomotion speed as well as ants’ capacity to navigate through crevices of varying sizes and the amount of load they can carry. May also indicate the type of diet, as ants with shorter RelHL tend to be predators.	[31,33,34]

### 2.3. Functional Traits

Five functional traits, computed using measurements taken by AMF under a microscope with a calibrated eyepiece micrometer, were selected following Liu et al. [35] (Table 1): Weber’s length (WL); relative eye length (RelEL), calculated as the ratio of EL to WL; relative scape length (RelSL), calculated as the ratio of SL to WL; relative mandible length (RelML), the ratio of ML to WL; and relative hindleg length (RelHL), calculated as the sum of HFL and HTL, divided by WL. The mean of the trait values for each species were used for functional diversity analyses. Other measurements, namely head length (HL) and width (HW), eye width (EW), and pronotum width (PW), were also taken but were not included due to their high correlation and ecological redundancy to our five selected traits. A maximum of three worker ants per species were measured for each elevational band. For singletons and doubletons, only one and two ants were measured, respectively. For species with worker polymorphism, only minors were measured. Ant species with no eyes are likely to be subterranean species [31] with ecological characteristics inherently different from species foraging above ground. Thus, the proposed justification for the use of eye size listed in Table 1 probably does not apply to subterranean species, and assigning a RelEL of 0 for these species may therefore mischaracterize the “functional position” of eyeless ants. We therefore removed these species from the analysis.

A total of 1,165 individuals (Mengla: 995; Ailaoshan: 170) were measured and used for computing functional alpha and beta diversity. We decided not to include Lijiang in our functional diversity analyses due to its small sample size (discussed in more detail in 2.4), and a species from Mengla (*Dorylus laevigatus* (Smith)) was removed because the only available specimens consisted of non-minor workers.

### 2.4. Data Analysis

As abundances of ants collected by different sampling methods represent different aspects (e.g., pitfall traps representing activities of ground foraging ants, litter extraction representing ant density, hand collection representing incidence of ant species), abundance, and abundance-based diversity indices were avoided, and incidence per plot was used for analyses. We used species richness as a measure of taxonomic alpha diversity, while functional alpha diversity was quantified using two incidence-based indices: Petchey and Gaston’s FD (hereafter referred to as FD; [36]) and functional richness or FRic [37]. FD is a dendrogram-based index wherein functional diversity is computed as the sum of the branch lengths of a dendrogram generated from the trait distance matrix for a community [12]. Originating from the convex hull model by Cornwell et al. [38], FRic defines functional diversity as the multi-dimensional volume (or range, if only one trait is considered) of one or more traits occupied by a given species assemblage. Its perimeter is determined by the most extreme points, making FRic sensitive to species with the smallest and largest trait values [12,37]. While the two indices are known to be highly correlated, FRic has a higher sensitivity to community assembly rules compared to FD. FD is generally correlated with species richness but is not sensitive to species splitting, or the splitting of one taxonomic species into two functionally similar species, unlike other indices [39]. To control for differences in the magnitude of measurements as well as trait redundancy and covariation, we first log-transformed the trait values to achieve a normal distribution, and then scaled and subjected the values to principal component analysis (PCA) [40]. As PCA requires the number of species to be more than the number of traits (we used five traits), we did not calculate FD and FRic for Lijiang where we collected a total of three species. We selected the axes which explained at least 90% of the trait variation. For calculating FD, we computed the multivariate Euclidean distances between all species to create a distance matrix. We then used the distance matrix to generate a trait dendrogram based on the Unweighted Pair Group Method with Arithmetic Mean (UPGMA) for hierarchical clustering. The normalized trait values were also used for FRic. FD and FRic were calculated using the R packages “picante” and “FD”, respectively [41,42].

We generated multi-linear models to explain the relationship between taxonomic (species richness) and functional alpha diversity (FD and FRic) with respect to elevation. For all three models, we included location (i.e., elevational transect) as a fixed factor as they explained more variation and had lower Akaike Information Criterion (AIC) scores than models without location. To test whether species richness, FD, and FRic showed a linear or logarithmic relationship with elevation, we ran models with log-transformed or untransformed elevation as fixed effects (location was excluded from this comparison) and compared the results using their AIC scores. Species richness and FD were untransformed while FRic was cube-root transformed to achieve data normality.

To test the relationship between mean daily understory temperature and elevation, we generated a simple linear regression using published [43] and unpublished understory temperature measurements recorded on 11 July to 8 September 2015 at the same plots from which ants were sampled in all three elevational transects. Hourly temperature was recorded from each plot using thermologgers (DS1923 Hygrochron® iButton®, Maxim, CA, USA) placed 1.3 m above the ground [43].

We calculated taxonomic and functional beta diversity using the Sørensen dissimilarity index (β_SOR_), which quantified pairwise species and functional dissimilarity between plots within each transect [44]. For functional beta diversity, we first generated multivariate trait space using principal coordinates analysis (PCoA), which calculated orthogonal axes representing different spectra of functional characteristics. The first and second PCoA axes explained 98.64% of the variation in Mengla and 97.68% of the variation in Ailaoshan, and, accordingly, were used for computing functional beta diversity. Ant species found in each plot were then projected onto the multivariate space as a convex hull. The Sørensen dissimilarity values were calculated based on the volumes of multivariate trait space shared by two sites and unique to each. The Sørensen dissimilarity values were plotted against differences in elevation between the same pairs of plots. The dissimilarity values were also decomposed into nestedness-resultant dissimilarity (β_SNE_), and turnover-resultant dissimilarity, also known as the Simpson index (β_SIM_) [45]. We then tested the relationship between taxonomic and functional beta diversity and elevational distance using Mantel tests with 4999 permutations. The taxonomic and functional beta diversity metrics were calculated using the R package “betapart” [44], and Mantel tests were conducted using the R package “vegan” [46].

As functional diversity tends to be positively correlated with the number of species, we generated null models to remove biases due to differences in species richness and make the values comparable across sites [40]. To generate null models for functional alpha diversity, we performed 999 constrained randomizations of the community data matrix using the independent swap method [47]. In unconstrained null models, the community data matrix is randomized so that other components of the data (e.g., species occupancy rates) aside from the component of interest vary among different models, thus increasing the chances of Type I error. The independent swap method, however, randomizes the community data matrix while maintaining the original species richness and species occupancy rate [40]. We calculated standardized effect sizes (SES) for each FD and FRic value and generated their observed ranks, which indicate where the observed SES values lie within the overall null probability distribution [40]. We then used SES values and their respective ranks to determine whether the observed FD and FRic values were significantly different from the null. Negative SES values with observed ranks lower than or equal to 25 (two-tailed test; α = 0.025) indicate that the observed FD or FRic is significantly lower than the mean expected value (i.e., trait clustering), while positive SES values with observed ranks greater than or equal to 975 (α = 0.025) indicate that the observed FD or FRic is significantly higher than the mean expected value (i.e., trait over-dispersion). SES-FD was computed using R package “picante”, while SES-FRic was computed using the R script by Swenson [40]. All analyses were conducted in R version 3.5.2.

## 3. Results

We recorded a total of 263 species from the three transects: 220 in Mengla, 55 in Ailaoshan, and three in Lijiang. Though there were no species shared among all transects or between Lijiang and Mengla, one was observed in both Lijiang and Ailaoshan and 14 in both Ailaoshan and Mengla. Mengla had the highest percentage of species present in all elevational bands (Mengla: 17%; Ailaoshan: 5%; Lijiang: 0%) and the highest number of species occurring in two adjacent elevational bands (Mengla: 53; Ailaoshan: 13; Lijiang: 1). Almost 30% (65) of the species in Mengla and more than half (34 species, 62%) of the species in Ailaoshan were restricted to one elevational band. Many species in Mengla and Ailaoshan were singletons, found in a single plot in the entire transect (Mengla: 47; Ailaoshan: 22). Of the 263 species, 12 eyeless species from Mengla and two eyeless species from Ailaoshan were excluded from functional diversity analyses. Incidence data and mean functional trait values for species recorded in Mengla, Ailaoshan, and Lijiang with eye length >0 are provided as Supplementary Materials (Tables S1–S6)

**Figure 1:**
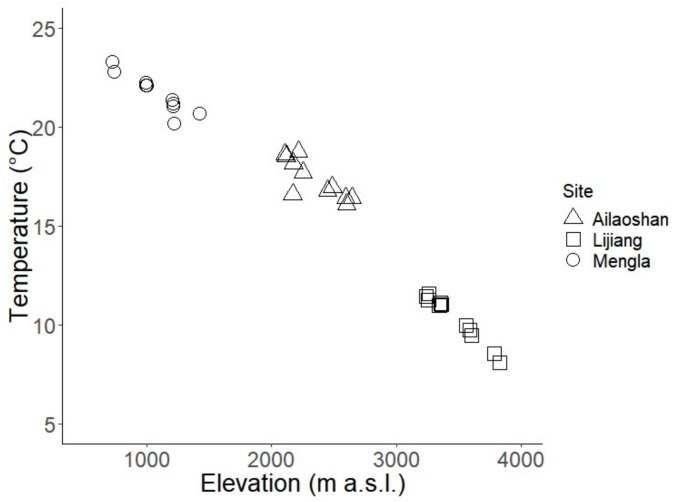
Mean daily understory temperatures from July to September 2015. Temperature data were recorded from each plot every two hours.

**Figure 2:**
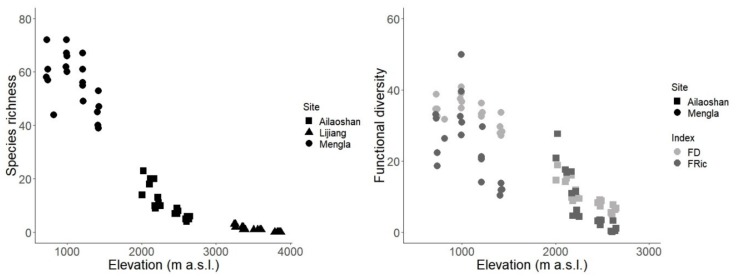
Taxonomic (**left**) and functional (**right**, showing FD in light grey and FRic in dark grey symbol) alpha diversity in Mengla (circle), Ailaoshan (square) and Lijiang (triangle) against actual elevation (in m a.s.l.), computed using species with eye length >0. Note the difference in x-axis values, as Lijiang was excluded from functional diversity analyses.

Mean daily understory air temperature decreased linearly with increasing elevation (*F*_1,30_ = 775.3, adjusted *R*^2^ = 0.962, *p* < 0.001) (Figure 1). Based on the linear models, species richness showed a significant decrease with elevation (*F*_3,56_ = 392.3, adjusted *R*^2^ = 0.952, *p* < 0.001). FD and FRic also decreased with elevation (FD: *F*_2,37_ = 431.4, adjusted *R*^2^ = 0.957, *p* < 0.001; FRic: *F*_3,36_ = 108.8, adjusted *R^2^* = 0.892, *p* <0.001; Figure 2). We additionally tested whether species richness, FD, and FRic showed a linear or logarithmic relationship with elevation. For species richness, the AIC score was lower when elevation was log-transformed (AIC = 420.88) than when elevation was untransformed (AIC = 449.73). In contrast, the AIC score was higher for FD and FRic when elevation was log-transformed (AIC_FD_ = 249.50; AIC_FRic_ = 62.21) than when elevation was untransformed (AIC_FD_ = 225.73; AIC_FRic_ = 49.57).

**Figure 3:**
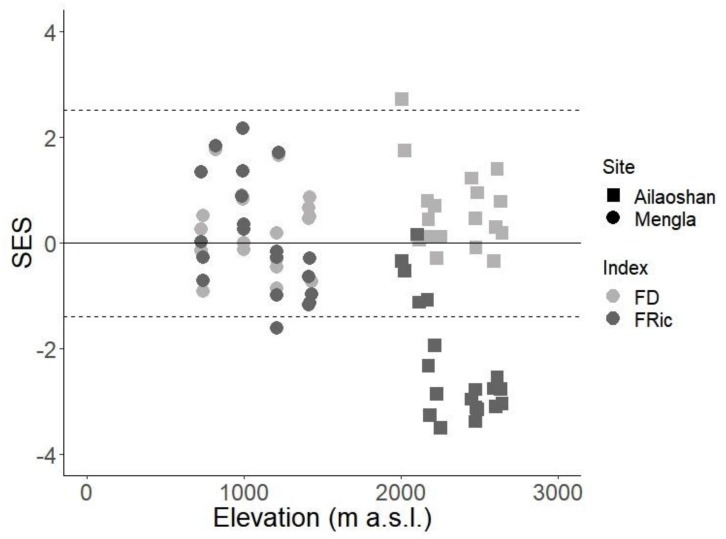
Standardized effect sizes (SES) for FD (light grey) and FRic (dark grey) in Mengla (circle) and Ailaoshan (square) against elevation (in m a.s.l.). Points above (for positive values) and below (for negative values) the dashed lines have SES values significantly different from the null model.

SES-FD and SES-FRic values in Mengla did not show distinct patterns, with only one plot having a significantly lower SES-FRic value from the mean expected value (Figure 3). In Ailaoshan, all plots found at the 2200, 2400, and 2600 m a.s.l. bands showed significantly lower SES-FRic values, while one plot in the 2000 m a.s.l. band showed a significantly high SES-FD value; all other SES-FD values as well as SES-FRic values from plots found in the 2000 m a.s.l. band did not differ significantly from expected.

**Figure 4:**
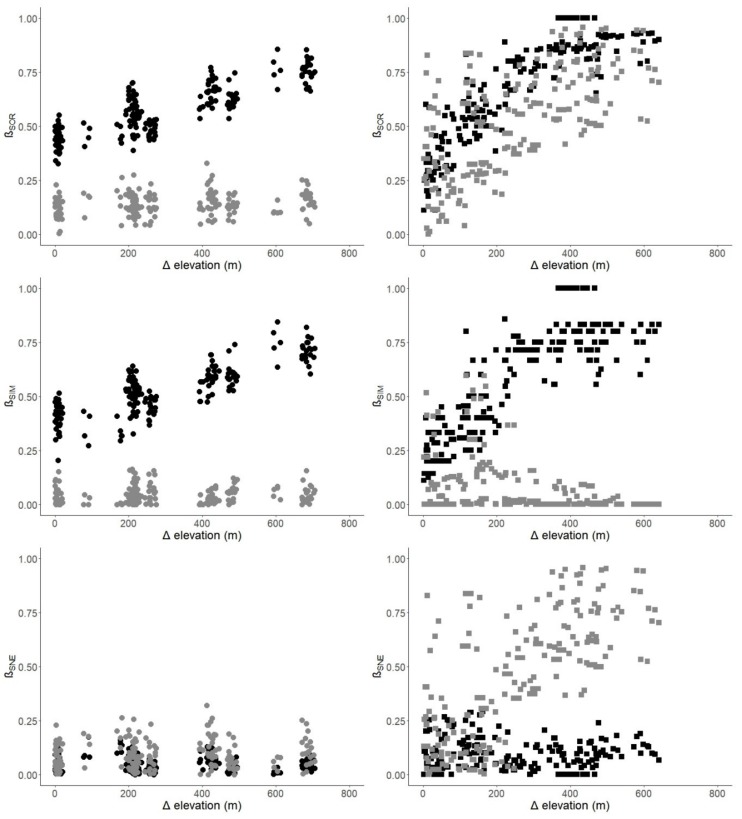
Pairwise taxonomic (black) and functional (gray) dissimilarities versus elevational distances between pairs of plots in Mengla (left, circles) and Ailaoshan (right, squares). β_SOR_ = Sørensen dissimilarity index or total dissimilarity; β_SIM_ = Simpson index or turnover-resultant dissimilarity; β_SNE_ = nestedness-resultant dissimilarity.

Taxonomic dissimilarity (β_SOR_) showed a significantly positive correlation with elevational distance in both Mengla and Ailaoshan (Table 2; Figure 4). When the dissimilarity values were decomposed into turnover-resultant dissimilarity (β_SIM_) and nestedness-resultant dissimilarity (β_SNE_), β_SIM_ showed a significantly positive correlation with elevational distances in Mengla and Ailaoshan, while β_SNE_ did not. Functional dissimilarity (β_SOR_) also showed a significantly positive correlation with elevational distance in both Mengla and Ailaoshan, but the correlation was weaker in Mengla. Unlike taxonomic dissimilarity, functional β_SIM_ did not show a significant correlation, and functional β_SNE_ was significantly and positively correlated with elevational distance in Ailaoshan (Table 2; Figure 4).

## 4. Discussion

**Figure A2:**
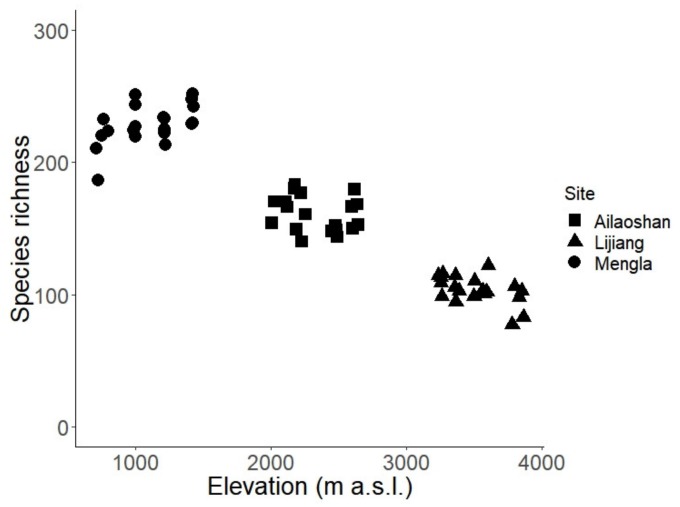
Adjusted moth species richness against elevation (in m a.s.l.) in Mengla, Ailaoshan and Lijiang. Species richness was adjusted based on the smallest sample size. Data from Ashton et al. [24].

Among the three transects, species richness was highest in tropical Mengla and lowest in subalpine Lijiang, following the latitudinal diversity gradient or LDG. Only Mengla showed a mid-elevation peak in species richness based on elevational bands. While the negative relationship between species richness and elevation observed in Ailaoshan and Lijiang may seem to contradict the general pattern of mid-elevation peaks in ant diversity [22], it is possible that the patterns we observed are due to the limited elevational ranges of our transects [48]. When the three transects were combined, ant species richness showed a general logarithmic decrease with increasing elevation. This pattern of decline, however, differed from that observed in moths collected from the same study sites by Ashton et al. [24], who reported a linear decrease in moth species richness (Figure A2 in Appendix A). Another study on latitudinal and elevational gradients of fig wasp and phytophage diversity within southwest China also showed highest species richness in the tropics and linearly decreasing trends with increasing elevation and latitude [49]. This suggests that taxonomic ant diversity is more sensitive to latitudinal and elevational gradients in the region. 

Our results showed a linear decrease in FD and FRic with increasing elevation. SES-FD and SES-FRic values in Mengla indicated that patterns of functional alpha diversity in our tropical transect were similar to what would be expected by chance, and thus were likely to be due to stochastic rather than deterministic processes. However, SES-FRic values in plots in Ailaoshan found above the 2000 m a.s.l. band were indicative of trait clustering. This, together with the non-linear decrease in species richness with elevation despite a linear decrease in mean hourly temperatures, implies that the thermal limit for ants in the region might be close to the temperature ranges in the higher elevations of the Ailaoshan transect, where ant communities are likely subject to environmental filtering. While we acknowledge that other factors aside from temperature may have shaped our observations, we also suspect that the inclusion of other functional traits, specifically physiological and behavioral traits related to thermal tolerance, would produce more accurate insights into functional responses of ants [22]. Incidentally, examining how insect community structure changes along elevational gradients may also provide insights on how insects might respond to future changes in climatic conditions because of the predictable relationship between elevation and temperature [48]. As more studies focus on determining which traits best represent adaptations and responses to environmental gradients, we expect functional diversity indices to better reflect which kind of processes shape community structure, and functional diversity in general to be more useful in predicting future distribution patterns.

Unlike alpha diversity, different patterns were found between taxonomic and functional beta diversities. As we hypothesized, taxonomic beta diversity in both Mengla and Ailaoshan increased with increasing elevational distance and was driven by species turnover (β_SIM_), as reflected by the high percentage of species restricted to only few elevational bands in both transects. In contrast, functional beta diversity patterns varied between Mengla (where both turnover- and nestedness-resultant functional dissimilarity did not change with increasing elevational distance) and Ailaoshan (where nestedness-resultant functional dissimilarity increased with elevational distance). The results for taxonomic beta diversity are similar to those in Bishop et al. [15], who reported a linear increase in β_SIM_ with elevational distance in southern African ant assemblages. However, in their study functional turnover was dominant at the lower half of the elevational gradient and functional nestedness at the higher half. They attributed functional turnover to the introduction of species that appeared to be specialized for predation and living in open areas, and functional nestedness to a loss of the most extreme traits at higher elevations. Our results imply that species with extreme trait values were replaced in high-elevation plots in the Ailaoshan transect, and so species turnover would cause the functional trait space to shrink in higher elevations and become a subset of that of plots in lower elevations.

Studies on elevational gradients of diversity have generally focused on a single transect (e.g., [15,17,23]). Here we incorporated three elevational transects to investigate how elevational diversity gradients change among the three locations at different latitudes, and found that elevation strongly influenced our observed diversity patterns. However, the effects of elevation and latitude are confounded in our study as the elevational ranges of the three transects were not fully crossed (i.e., elevations did not overlap among the transects). We cannot readily separate the effects of these two predictors, as elevations in Yunnan Province are generally lower in the south (viz. tropical Mengla) and increase towards the north (through subtropical Ailaoshan to subalpine Lijiang). More studies on elevational diversity gradients involving mountain systems in different latitudes and biomes are therefore needed in order to explicitly test how elevation, latitude, and other environmental factors contribute to observed patterns of montane biodiversity. Such efforts have been made for the taxonomic diversity of geometrid moths [6], where standardized sampling was conducted across multiple elevational studies at a global scale. Global distributions of ant assemblages are currently being collated using an online database which not only includes taxonomic information but trait measurements of individual ants as well [50]. We envisage that global patterns of ant diversity will soon be revealed with this kind of concerted effort.

## 5. Conclusions

Taxonomic diversity of ants decreased logarithmically with increasing elevation in southwest China despite temperature and functional diversity decreasing linearly. Community structure in Ailaoshan at plots found above the 2000 m a.s.l. band was explained by deterministic processes, while stochastic processes appear to have shaped community structure in Mengla. The differences in our results and the linear decrease reported for moth species richness in the same elevational transects imply that abiotic factors related to elevation affect community assemblages of different insect taxa to varying degrees. Though taxonomic beta diversity increased with increasing elevational distance in both tropical Mengla and subtropical Ailaoshan, functional beta diversity patterns differed between the two sites. Our results reflect ants’ preference for warmer climates, and suggest that the temperature in the higher elevations of the Ailaoshan transect is close to their thermal limits, causing the functional trait space to shrink at higher elevations and to become a subset of plots in lower elevations. Aside from providing data on ant diversity in southwest China, this study is also the first in the region to combine both taxonomic and functional approaches in examining insect diversity along elevational gradients. The results of our study contribute to the growing knowledge on latitudinal and elevational diversity gradients for insects.

## Figures and Tables

**Table 2:** Mantel correlations between beta diversity indices and elevational distance. β_SOR_ = Sørensen dissimilarity index or total dissimilarity; β_SIM_ = Simpson index or turnover-resultant dissimilarity; β_SNE_ = nestedness-resultant dissimilarity. Correlations with significant *p*-values (in parentheses) are shown in bold.

Index	Mengla		Ailaoshan	
Taxonomic β_SOR_	**0.826**	**(<0.001)**	**0.873**	**(<0.001)**
β_SIM_	**0.813**	**(<0.001)**	**0.833**	**(<0.001)**
β_SNE_	−0.044	(0.690)	−0.088	(0.893)
Functional β_SOR_	**0.187**	**(0.017)**	**0.655**	**(<0.001)**
β_SIM_	0.096	(0.123)	−0.347	(1)
β_SNE_	0.088	(0.133)	**0.709**	**(<0.001)**

## Supplementary Materials

The following are available online at https://www.mdpi.com/2075-4450/10/5/128/s1.
