# Supplementary material for: Taxonomic and Functional Ant Diversity Along tropical, Subtropical, and Subalpine Elevational Transects in Southwest China
Source: Insects. 2019 May 3;10(5):128. doi: 10.3390/insects10050128 (PMC6572390; doi:10.3390/insects10050128)
Supplement: Supplementary file 1 [file insects-10-00128-s001.pdf]

Table 1. Mean Weber's length (WL), relative eye length (RelEL), relative scape length (RelSL), relative mandible length (RelML) and relative hindleg length (RelHL) of species/morphospecies in Lijiang.

Species/morphospecies	WL	RelEL	RelSL	RelML	RelHL
Myrmica pleiorhytida	1.97	0.13	0.65	0.40	1.38
Temnothorax cf. *striatus*	0.84	0.17	0.69	0.39	1.19
Temnothorax wui	0.91	0.19	0.61	0.37	1.18

Table 2. Mean Weber's length (WL), relative eye length (RelEL), relative scape length (RelSL), relative mandible length (RelML) and relative hindleg length (RelHL) of species/morphospecies in Ailaoshan with eye length >0.

Species/morphospecies	WL	RelEL	RelSL	RelML	RelHL
Amblyopone amblyops	1.02	0.03	0.43	0.57	0.98
Amblyopone awa	1.48	0.04	0.39	0.54	0.96
Amblyopone octodentata	1.23	0.03	0.40	0.54	0.90
Amblyopone triloba	1.48	0.03	0.46	0.58	0.93
Brachyponera luteipes B	1.54	0.11	0.68	0.38	1.37
Camponotus sp. A	1.65	0.21	0.70	0.33	1.61
Camponotus sp. B	2.55	0.21	0.71	0.25	1.57
Carebara obtusidenta	0.43	0.05	0.62	0.48	1.08
Cerapachys biroi	0.72	0.04	0.36	0.25	0.89
Cerapachys sulcinodis	1.56	0.10	0.44	0.34	1.13
Crematogaster inflata	1.02	0.18	0.63	0.53	1.41
Ectomomyrmex javana	3.00	0.08	0.58	0.44	1.21
Gaoligongidris planodorsa	0.83	0.11	0.53	0.48	1.13
Gnamptogenys coccina	1.68	0.07	0.58	0.38	1.16
Hypoponera nippona	0.89	0.03	0.55	0.38	1.04
Hypoponera truncata	1.11	0.05	0.57	0.38	1.11
Lasius draco	1.73	0.13	0.74	0.44	1.71
Monomorium gracillimum	0.52	0.02	0.63	0.46	1.13
Monomorium pharaonis	0.72	0.14	0.72	0.36	1.33
Myrmecina guangxiensis	0.88	0.08	0.65	0.45	1.13
Myrmecina striata	0.89	0.08	0.68	0.46	1.14
Myrmica margaritae	1.58	0.14	0.73	0.41	1.43
Myrmica ritae	1.69	0.14	0.77	0.37	1.50
Myrmica sp. A	1.78	0.14	0.79	0.38	1.54
Myrmica sp. C	2.63	0.13	0.68	0.39	1.68
Myrmica titanica	2.89	0.12	0.73	0.38	1.71
Nylanderia sp. B	0.84	0.19	0.90	0.41	1.71
Nylanderia vividula	0.78	0.15	0.91	0.41	1.64
Nylanderia yerburyi	0.73	0.19	0.89	0.37	1.71
Perissomyrmex fissus	0.97	0.10	0.81	0.52	1.58
Pheidole indosinensis	0.85	0.14	0.81	0.48	1.49
Pheidole magna	1.15	0.13	0.83	0.50	1.79
Ponera cf. *pianmana*	1.16	0.02	0.55	0.37	0.97
Ponera menglana	1.14	0.04	0.55	0.39	1.00
Ponera pianmana	0.77	0.03	0.52	0.39	0.91
Prenolepis sp. A	1.17	0.18	0.91	0.42	1.95
Proceratium longigaster	0.98	0.02	0.55	0.41	1.09
Stenamma jeriorum	0.92	0.13	0.55	0.42	1.12
Stigmatomma meiliana	1.60	0.03	0.43	0.53	1.00
Strumigenys cf. *phasma*	0.51	0.02	0.47	0.24	1.08
Strumigenys paraposta	0.91	0.07	0.49	0.43	1.16
Tapinoma geei	0.96	0.17	0.71	0.29	1.48
Temnothorax cf. *brevispinus*	0.68	0.19	0.67	0.38	1.24
Temnothorax cf. *maorensis*	0.83	0.18	0.66	0.33	1.28
Temnothorax cf. *pisarskii*	0.84	0.17	0.67	0.38	1.26
Temnothorax cf. *ruginosus*	0.78	0.16	0.72	0.40	1.25
Temnothorax cf. *striatus*	0.60	0.19	0.72	0.41	1.21
Temnothorax hengshanensis	1.00	0.16	0.66	0.40	1.34
Temnothorax orchidus	0.94	0.17	0.60	0.38	1.23
Temnothorax sp.	0.58	0.19	0.76	0.41	1.21
Tetramorium aptum	0.88	0.16	0.57	0.45	1.18

Tetramorium khnum	0.86	0.16	0.58	0.49	1.21
Vollenhovia cf. *emeryi*	1.02	0.14	0.47	0.35	0.96

Table 3. Mean Weber's length (WL), relative eye length (RelEL), relative scape length (RelSL), relative mandible length (RelML) and relative hindleg length (RelHL) of species/morphospecies in Mengla with eye length > 0.

Species/morphospecies	WL	RelEL	RelSL	RelML	RelHL
Acropyga yaeyamensis	0.98	0.12	0.81	0.49	1.72
Amblyopone awa	1.10	0.04	0.40	0.45	0.87
Amblyopone crenata	2.00	0.05	0.44	0.55	1.11
Anochetus cf. *subcoecus*	1.53	0.07	0.59	0.43	1.28
Anochetus subcoecus	1.21	0.06	0.63	0.47	1.22
Aphaenogaster beccarii	1.84	0.12	0.98	0.34	2.09
Aphaenogaster exasperata	2.50	0.12	0.81	0.33	1.97
Aphaenogaster feae	2.02	0.13	0.93	0.35	2.12
Aphaenogaster lepida	2.00	0.12	0.82	0.35	1.88
Aphaenogaster schurri	2.13	0.14	0.91	0.37	2.05
Brachyponera luteipes C	1.37	0.10	0.64	0.40	1.30
Brachyponera luteipes D	1.82	0.11	0.66	0.37	1.40
Brachyponera luteipes I	1.61	0.08	0.66	0.34	1.35
Brachyponera luteipes K	1.69	0.07	0.65	0.40	1.36
Brachyponera luteipes L	1.24	0.11	0.61	0.40	1.25
Camponotus cf. *wroughtoni*	1.55	0.19	0.71	0.26	1.42
Camponotus crassiquamis	2.75	0.16	0.85	0.27	1.95
Camponotus holosericeus	4.05	0.12	0.79	0.26	1.98
Camponotus invidus	2.83	0.16	0.74	0.23	1.52
Camponotus lasiselene	1.42	0.16	0.68	0.28	1.42
Camponotus leonardi	2.05	0.21	0.68	0.28	2.10
Camponotus marginatus	2.50	0.18	0.75	0.23	1.58
Camponotus mitis	3.68	0.14	0.94	0.22	2.07
Camponotus nicobarensis	2.36	0.17	0.81	0.27	1.90
Camponotus politae	1.60	0.19	0.97	0.22	2.03
Camponotus radiatus	1.30	0.23	0.58	0.23	1.31
Camponotus rothneyi	2.10	0.17	0.67	0.26	2.19
Camponotus singularis	5.00	0.13	0.90	0.27	2.11
Camponotus sp. H	2.48	0.18	0.77	0.22	1.54
Camponotus sp. K	1.70	0.19	0.72	0.26	1.56
Camponotus sp. L	1.35	0.19	0.70	0.26	1.41
Camponotus vitreus	1.75	0.21	0.77	0.26	1.93
Cardiocondyla wroughtonii	0.49	0.19	0.63	0.37	1.16
Carebara acutispina	0.40	0.03	0.55	0.50	1.00
Carebara affinis	0.75	0.07	0.65	0.46	1.54
Carebara altinoda	0.45	0.04	0.65	0.46	1.19
Carebara obtusidenta	0.33	0.03	0.61	0.50	1.09
Carebara rectidorsa	0.34	0.03	0.61	0.45	1.09
Carebara reticapita	0.31	0.03	0.59	0.47	0.94
Carebara trechiderus	0.79	0.07	0.71	0.50	1.61
Cataulacus granulatus	1.37	0.32	0.43	0.28	1.16
Cerapachys longitarsus	1.25	0.18	0.28	0.30	1.00
Cerapachys sulcinodis	2.08	0.13	0.45	0.35	1.22
Crematogaster dalyi	0.99	0.19	0.68	0.44	1.52
Crematogaster ebenina	0.95	0.20	0.70	0.41	1.58
Crematogaster ferrarii	0.82	0.18	0.64	0.43	1.45
Crematogaster hodgsoni	0.94	0.19	0.66	0.43	1.40
Crematogaster macaoensis	1.08	0.19	0.70	0.33	1.69
Crematogaster matsumurai	0.78	0.18	0.62	0.38	1.28
Crematogaster millardi	0.66	0.18	0.58	0.39	1.20
Crematogaster osakensis	0.63	0.20	0.70	0.42	1.42

Crematogaster rogenhoferi	0.92	0.20	0.71	0.39	1.66
Crematogaster subnuda	1.01	0.20	0.76	0.36	1.71
Crematogaster wroughtoni	1.03	0.20	0.89	0.41	1.83
Crematogaster zoceensi	0.80	0.21	0.78	0.45	1.62
Cryptopone sauteri	2.37	0.06	0.49	0.44	0.98
Diacamma rugosum	3.72	0.13	0.74	0.37	1.52
Dilobocondyla gasteroreticulatus	1.56	0.17	0.45	0.35	1.03
Discothyrea banna	0.76	0.13	0.70	0.39	1.34
Discothyrea diana	0.57	0.06	0.59	0.35	1.12
Dolichoderus affinis	1.27	0.19	0.73	0.36	1.84
Dolichoderus feae	1.97	0.17	0.72	0.33	2.07
Dolichoderus incisus	1.26	0.18	0.72	0.35	1.87
Dolichoderus moggridgei	0.90	0.23	0.73	0.38	1.73
Dolichoderus squamanodus	1.10	0.20	0.64	0.39	1.44
Dolichoderus taprobanae	0.83	0.23	0.74	0.40	1.69
Dolichoderus thoracius	1.19	0.19	0.67	0.37	1.69
Echinopla cherapunjiensis	2.01	0.17	0.73	0.30	1.48
Ectomomyrmex annamita	2.10	0.07	0.52	0.38	0.98
Ectomomyrmex javana	3.39	0.08	0.57	0.40	1.21
Ectomomyrmex leeuwenhoeki	2.58	0.09	0.55	0.40	1.10
Ectomomyrmex zhengi	4.24	0.09	0.59	0.41	1.26
Emeryopone melaina	1.56	0.05	0.63	0.51	1.00
Euponera pilosior	1.56	0.03	0.45	0.39	1.03
Gauromyrmex cf. *acanthinus*	0.65	0.17	0.52	0.29	1.07
Gnamptogenys bicolor	1.66	0.12	0.58	0.36	1.20
Hypoponera cf. *sauteri*	0.56	0.04	0.48	0.34	0.91
Hypoponera confinis	1.05	0.04	0.59	0.38	1.17
Hypoponera nippona	0.95	0.04	0.57	0.35	1.21
Hypoponera sauteri	0.72	0.04	0.56	0.35	1.06
Hypoponera truncata	1.35	0.06	0.59	0.38	1.16
Kartidris sparsipila	1.20	0.17	0.69	0.42	1.64
Leptogenys birmana	2.68	0.10	0.53	0.41	1.25
Leptogenys kitteli	2.93	0.12	0.67	0.32	1.45
Leptogenys kraepelini	3.13	0.13	0.64	0.28	1.48
Leptogenys pangui	4.70	0.15	0.74	0.24	1.68
Leptogenys processionalis	1.90	0.09	0.53	0.37	1.16
Liometopum sinense	1.37	0.17	0.69	0.35	1.66
Lophomyrmex quadrispinosus	0.90	0.16	0.74	0.44	1.59
Meranoplus laeviventris	1.10	0.18	0.69	0.43	1.53
Mesoponera melanaria	2.70	0.07	0.60	0.44	1.34
Monomorium destructor	0.56	0.05	0.65	0.40	1.30
Monomorium latinode	0.64	0.15	0.74	0.45	1.44
Monomorium orientale	0.45	0.13	0.65	0.38	1.09
Monomorium pharaonis	0.69	0.13	0.74	0.36	1.30
Myopias conicara	2.93	0.11	0.49	0.47	1.02
Myrmecina curvispina	1.00	0.12	0.72	0.44	1.24
Myrmecina guangxiensis	0.67	0.08	0.65	0.46	1.08
Myrmecina sinensis	0.68	0.06	0.74	0.50	1.03
Myrmecina striata	0.80	0.08	0.60	0.47	1.01
Mystrium oculatum	1.36	0.03	0.59	0.88	1.17
Nylanderia birmana	1.20	0.17	0.90	0.36	1.82
Nylanderia flavipes	0.77	0.13	0.92	0.38	1.72
Nylanderia vividula	0.56	0.17	0.88	0.42	1.57
Odontomachus circulus	3.97	0.09	0.83	0.54	1.70
Odontomachus monticola	3.55	0.08	0.78	0.51	1.56
Odontoponera transversa	3.78	0.11	0.57	0.36	1.33

Oecophylla smaragdina	3.20	0.17	0.91	0.39	2.30
Paraparatrechina sauteri	0.48	0.22	0.87	0.44	1.63
Pheidole cf. *acantha*	1.12	0.14	0.93	0.50	1.91
Pheidole cf. *quadricuspis*	0.88	0.16	0.91	0.48	1.68
Pheidole fervens	0.98	0.13	0.94	0.46	1.76
Pheidole fortis	1.01	0.15	0.88	0.49	1.78
Pheidole gatesi	1.60	0.14	0.88	0.49	2.07
Pheidole laevicolor	0.80	0.13	0.79	0.47	1.44
Pheidole ochracea	0.85	0.15	0.96	0.43	1.80
Pheidole parva	0.71	0.13	0.74	0.45	1.42
Pheidole pieli	0.56	0.16	0.72	0.47	1.33
Pheidole plagiaria	1.20	0.14	0.95	0.43	1.91
Pheidole rugithorax	0.91	0.15	0.89	0.43	1.71
Pheidole smythiesii	1.38	0.13	0.93	0.50	2.05
Pheidole tjibodana	0.69	0.14	0.70	0.50	1.33
Pheidole tumida	1.05	0.16	0.95	0.42	1.93
Pheidole vieti	0.74	0.10	0.78	0.49	1.57
Pheidole vulgaris	0.75	0.13	0.76	0.45	1.52
Pheidole watsoni	0.62	0.16	0.74	0.47	1.46
Plagiolepis alluaudi	0.46	0.20	0.78	0.35	1.33
Platythyrea parallela	2.02	0.10	0.45	0.26	1.00
Polyrhachis armata	3.64	0.15	0.89	0.25	2.44
Polyrhachis bicolor	2.25	0.19	0.92	0.29	2.14
Polyrhachis brevicorpa	2.15	0.23	0.93	0.33	2.28
Polyrhachis ceylonensis	2.15	0.20	0.94	0.29	1.97
Polyrhachis cf. *clypeata*	2.35	0.21	0.94	0.30	2.00
Polyrhachis cf. *tibialis*	2.19	0.18	0.93	0.28	1.80
Polyrhachis clypeata	2.30	0.22	0.72	0.28	1.52
Polyrhachis dentihumera	2.10	0.21	1.01	0.35	2.55
Polyrhachis halidayi	2.34	0.20	0.85	0.32	1.76
Polyrhachis illaudata	3.51	0.15	0.94	0.28	1.97
Polyrhachis moesta	1.87	0.21	0.97	0.29	1.89
Polyrhachis orbihumera	1.85	0.24	0.95	0.35	2.27
Polyrhachis phipsoni	2.15	0.19	0.94	0.28	2.21
Polyrhachis pubescens	2.15	0.16	0.95	0.28	1.86
Polyrhachis sp. C	4.30	0.16	0.93	0.27	2.14
Polyrhachis thompsoni	2.50	0.18	0.89	0.27	2.04
Polyrhachis thrinax	2.20	0.20	0.89	0.34	1.89
Polyrhachis tibialis	2.17	0.18	0.93	0.27	1.95
Ponera baka	0.60	0.03	0.47	0.37	0.82
Ponera cf. *diodonta*	0.76	0.04	0.50	0.34	0.89
Ponera longlina	0.82	0.04	0.54	0.38	0.97
Ponera menglana	0.90	0.04	0.57	0.40	0.97
Ponera nangongshana	0.76	0.04	0.54	0.38	1.02
Prenolepis emmae	1.05	0.21	0.88	0.38	1.74
Prenolepis magnocula	1.01	0.23	1.13	0.34	2.32
Prenolepis naoroji	1.13	0.21	1.04	0.34	2.14
Prenolepis sphingthorax	1.29	0.14	0.97	0.29	1.97
Pristomyrmex brevispinosus	1.09	0.14	0.95	0.53	1.76
Pristomyrmex hamatus	0.92	0.17	0.96	0.54	1.64
Proceratium cf. *longigaster*	1.50	0.05	0.56	0.40	1.23
Proceratium longigaster	0.74	0.03	0.57	0.41	1.06
Proceratium zhaoi	0.64	0.03	0.56	0.50	1.13
Pseudolasius silvestrii	0.84	0.12	0.77	0.44	1.67
Pseudoneoponera rufipes	5.10	0.10	0.51	0.40	1.20
Recurvidris recurvispinosa	0.67	0.17	0.65	0.39	1.27

Rhopalomastix rothneyi	0.66	0.17	0.32	0.33	0.97
Solenopsis indagatrix	0.44	0.09	0.59	0.41	1.02
Strumigenys assamensis	0.76	0.11	0.41	0.30	1.24
Strumigenys cf. *bryanti*	0.73	0.08	0.52	0.50	1.17
Strumigenys cf. *exhilirina*	0.70	0.09	0.49	0.37	1.03
Strumigenys doriae	1.20	0.05	0.50	0.50	1.38
Strumigenys mitis	0.43	0.12	0.49	0.32	1.16
Strumigenys nanzanensis	0.68	0.12	0.56	0.39	1.25
Strumigenys nongba	0.60	0.08	0.54	0.38	1.39
Strumigenys rallarhina	0.59	0.07	0.64	0.50	1.26
Strumigenys sauteri	0.61	0.08	0.40	0.13	1.02
Strumigenys strugax	0.68	0.08	0.46	0.43	1.10
Strumigenys sydorata	0.71	0.09	0.41	0.33	1.08
Strumigenys tritomea	0.83	0.10	0.44	0.36	1.25
Tapinoma indicum	0.46	0.22	0.70	0.43	1.43
Technomyrmex albipes	0.84	0.18	0.69	0.34	1.37
Technomyrmex antennus	1.29	0.16	0.79	0.27	1.58
Technomyrmex elatior	0.93	0.19	0.74	0.38	1.49
Technomyrmex kraepelini	1.36	0.16	0.79	0.30	1.61
Technomyrmex obscurior	1.32	0.16	0.79	0.26	1.59
Technomyrmex pratensis	1.02	0.20	0.66	0.26	1.36
Technomyrmex vitiensis	1.06	0.19	0.72	0.26	1.42
Technomyrmex yamanei	1.19	0.16	0.73	0.36	1.80
Temnothorax angulohumerus	0.83	0.17	0.69	0.39	1.39
Temnothorax cf. *congruus*	0.74	0.19	0.57	0.35	1.43
Temnothorax cf. *zhejiangensis*	0.66	0.21	0.55	0.29	1.18
Temnothorax sp. C	0.79	0.15	0.50	0.27	1.05
Tetramorium aptum	0.79	0.15	0.60	0.44	1.24
Tetramorium indosinense	1.01	0.16	0.68	0.50	1.51
Tetramorium kraepelini	0.62	0.19	0.55	0.47	1.13
Tetramorium lanuginosum	0.70	0.19	0.55	0.43	1.15
Tetramorium laparum	0.78	0.19	0.63	0.42	1.27
Tetramorium melleus	0.90	0.16	0.73	0.47	1.71
Tetramorium nipponense	1.03	0.19	0.57	0.39	1.21
Tetramorium obtusidens	0.78	0.19	0.54	0.36	1.12
Tetramorium pacificum	1.06	0.19	0.57	0.32	1.38
Tetramorium smithi	1.02	0.17	0.61	0.41	1.31
Tetramorium wroughtonii	0.80	0.18	0.67	0.50	1.49
Tetraponera allaborans	1.44	0.24	0.35	0.24	1.00
Tetraponera attenuata	2.13	0.18	0.36	0.24	1.03
Tetraponera furcata	1.27	0.26	0.35	0.24	1.01
Vollenhovia emeryi	0.74	0.16	0.48	0.36	0.96
Vollenhovia lucimandibula	1.08	0.10	0.49	0.43	1.00
Vombisidris tibeta	1.01	0.18	0.53	0.35	1.13

Table 4. Incidence data of species/morphospecies with eye length >0 in each study plot in Lijiang.

Species/morphospecies	3200					3400					3600				
	3240	3254	3258	3259	3256	3346	3355	3361	3365	3390	3605	3588	3559	3559	3504
Myrmica pleiorhytida	X	X	X	X	X	X	X		X	X	X	X	X	X	X
Temnothorax cf. *striatus*	X	X	X	X	X	X	X	X	X						
Temnothorax wui	X	X		X	X										

Table 5. Incidence data of species/morphospecies with eye length >0 in each study plot in Ailaoshan.

Species/morphospecies	2000					2200					2400					2600				
	2170	2120	2107	2024	2006	2219	2177	2253	2186	2229	2450	2489	2480	2477	2475	2596	2604	2646	2636	2615
Amblyopone amblyops		X																		
Amblyopone awa														X						
Amblyopone octodentata				X	X															
Amblyopone triloba																			X	
Brachyponera luteipes B	X		X	X																
Camponotus sp. A				X																
Camponotus sp. B							X													
Carebara obtusidenta	X	X	X	X	X	X	X													
Cerapachys biroi				X																
Cerapachys sulcinodis	X		X	X	X	X				X										
Crematogaster inflata				X																
Ectomomyrmex javana	X	X	X	X	X	X														
Gaoligongidris planodorsa	X	X	X	X	X															
Gnamptogenys coccina	X					X		X		X										
Hypoponera nippona		X	X	X	X	X	X	X	X	X										
Hypoponera truncata		X	X	X		X	X	X		X										
Lasius draco	X																			
Monomorium gracillimum	X		X																	
Monomorium pharaonis										X										
Myrmecina guangxiensis		X	X	X	X		X		X		X			X	X	X	X	X	X	X
Myrmecina striata	X	X	X	X																
Myrmica margaritae						X														
Myrmica ritae					X							X			X	X				X
Myrmica sp. A	X	X				X					X					X		X		
Myrmica sp. C										X										
Myrmica titanica	X		X			X	X	X	X											
Nylanderia sp. B											X	X	X	X	X					
Nylanderia vividula	X	X		X	X	X	X	X	X	X										
Nylanderia yerburyi		X	X																	
Perissomyrmex fissus											X	X	X	X	X		X	X	X	X
Pheidole indosinensis	X	X	X	X	X	X	X	X	X	X	X	X	X	X	X					
Pheidole magna	X		X	X	X															
Ponera cf. *pianmana*	X	X	X			X	X	X	X	X										
Ponera menglana											X	X	X	X	X					
Ponera pianmana	X	X	X	X	X	X	X	X	X	X		X	X	X	X					
Prenolepis sp. A	X	X						X												
Proceratium longigaster	X	X												X	X					

Stenamma jeriorum												X				X	X	X	X	X
Stigmatomma meiliana																				X
Strumigenys cf. *phasma*					X															
Strumigenys paraposta		X	X	X																
Tapinoma geei				X																
Temnothorax cf. *brevispinus*	X		X	X																
Temnothorax cf. *maorensis*													X							
Temnothorax cf. *pisarskii*		X																		
Temnothorax cf. *ruginosus*								X			X	X	X	X	X	X	X	X	X	X
Temnothorax cf. *striatus*	X			X					X	X										
Temnothorax hengshanensis																		X		
Temnothorax orchidus		X																		
Temnothorax sp.									X											
Tetramorium aptum				X																
Tetramorium khnum				X	X															
Vollenhovia cf. *emeryi*		X																		

Table 6. Incidence data of species/morphospecies with eye length > in each study plot in Mengla.

Species/morphospecies	800					1000					1200					1400				
	725	741	818	728	739	997	999	995	995	985	1211	1212	1209	1219	1212	1412	1411	1421	1421	1430
Acropyga yaeyamensis	X																			
Amblyopone awa																		X		
Amblyopone crenata							X													
Anochetus cf. *subcoecus*	X																			
Anochetus subcoecus			X														X		X	
Aphaenogaster beccarii	X	X	X	X	X	X	X	X	X	X										
Aphaenogaster exasperata																				X
Aphaenogaster feae								X				X			X					
Aphaenogaster lepida																				X
Aphaenogaster schurri		X								X										
Brachyponera luteipes C	X	X	X	X	X	X	X	X	X	X	X	X	X	X	X	X	X	X		X
Brachyponera luteipes D		X		X		X		X		X							X	X	X	X
Brachyponera luteipes I	X					X		X		X			X				X	X	X	X
Brachyponera luteipes K																X	X		X	
Brachyponera luteipes L		X		X	X			X	X								X	X	X	X
Camponotus cf. *wroughtoni*												X								
Camponotus crassiquamis												X					X	X		
Camponotus holosericeus										X	X	X				X		X		
Camponotus invidus													X							
Camponotus lasiselene				X		X					X									
Camponotus leonardi		X						X	X											
Camponotus marginatus																		X		X
Camponotus mitis	X	X	X	X	X	X		X	X	X					X					X
Camponotus nicobarensis				X			X	X				X								
Camponotus politae														X						
Camponotus radiatus																	X			
Camponotus rothneyi			X	X		X														
Camponotus singularis	X	X		X			X		X	X	X							X		
Camponotus sp. H												X								X
Camponotus sp. K										X					X					
Camponotus sp. L																X				
Camponotus vitreus		X							X	X	X	X	X	X						
Cardiocondyla wroughtonii	X							X												

Carebara acutispina	X					X			X											
Carebara affinis	X	X		X	X			X	X											
Carebara altinoda	X					X	X			X	X	X				X		X		
Carebara obtusidenta	X																	X		X
Carebara rectidorsa			X	X	X	X	X	X	X	X			X	X	X	X	X	X		
Carebara reticapita	X	X	X	X	X	X	X	X	X	X	X	X	X	X	X	X	X	X	X	X
Carebara trechiderus	X	X		X	X			X			X	X	X	X	X		X	X	X	X
Cataulacus granulatus	X							X						X	X				X	
Cerapachys longitarsus								X												
Cerapachys sulcinodis							X				X			X		X	X	X	X	X
Crematogaster dalyi																X			X	
Crematogaster ebenina							X								X				X	
Crematogaster ferrarii			X		X		X			X	X	X	X	X	X			X		X
Crematogaster hodgsoni				X																
Crematogaster macaoensis	X																			
Crematogaster matsumurai					X															
Crematogaster millardi							X	X												
Crematogaster osakensis		X		X		X	X	X	X	X	X	X	X	X	X				X	
Crematogaster rogenhoferi	X	X		X	X			X			X									
Crematogaster subnuda											X	X								X
Crematogaster wroughtoni							X					X								
Crematogaster zoceensi								X	X											
Cryptopone sauteri				X		X			X	X			X		X	X		X		X
Diacamma rugosum																X		X		
Dilobocondyla gasteroreticulatus						X					X	X			X					
Discothyrea banna									X	X					X					
Discothyrea diana		X		X				X	X			X	X							
Dolichoderus affinis	X	X			X	X	X			X	X									
Dolichoderus feae			X							X										
Dolichoderus incisus	X	X				X	X		X	X	X	X	X	X	X	X	X		X	X
Dolichoderus moggridgei				X				X	X	X			X	X		X	X	X	X	X
Dolichoderus squamanodus						X					X	X	X	X		X	X			X
Dolichoderus taprobanae									X											
Dolichoderus thoracius											X			X	X			X		
Echinopla cherapunjiensis	X			X	X															
Ectomomyrmex annamita				X																
Ectomomyrmex javana		X	X	X	X	X	X	X	X		X	X	X	X	X	X	X	X	X	X

Ectomomyrmex leeuwenhoeki	X	X	X		X	X	X			X										
Ectomomyrmex zhengi	X					X		X	X	X			X	X	X	X		X	X	X
Emeryopone melaina										X										
Euponera pilosior					X							X								
Gauromyrmex cf. *acanthinus*					X				X		X									
Gnamptogenys bicolor	X	X	X	X				X					X	X	X					
Hypoponera cf. *sauteri*				X					X	X		X					X	X	X	
Hypoponera confinis					X				X			X					X			
Hypoponera nippona														X			X			X
Hypoponera sauteri	X	X	X		X	X	X	X	X	X	X	X	X	X	X	X	X	X		X
Hypoponera truncata	X	X	X	X			X	X												
Kartidris sparsipila			X	X	X		X	X			X	X		X	X			X		X
Leptogenys birmana				X						X										
Leptogenys kitteli		X					X	X							X	X	X	X		X
Leptogenys kraepelini						X	X			X			X			X	X			
Leptogenys pangui								X												
Leptogenys processionalis	X	X																		
Liometopum sinense	X																			
Lophomyrmex quadrispinosus											X	X	X	X	X	X	X	X	X	X
Meranoplus laeviventris		X		X				X		X										
Mesoponera melanaria				X	X					X		X								
Monomorium destructor	X		X	X	X	X			X											
Monomorium latinode	X	X	X	X	X	X	X	X	X	X	X	X	X	X	X					
Monomorium orientale			X	X	X	X	X	X	X	X		X	X		X			X		
Monomorium pharaonis	X				X	X		X		X			X	X	X					
Myopias conicara		X								X										
Myrmecina curvispina													X			X	X		X	
Myrmecina guangxiensis	X	X	X	X	X	X		X	X									X		
Myrmecina sinensis													X							
Myrmecina striata						X					X			X	X		X			
Mystrium oculatum	X		X		X	X		X	X	X				X						
Nylanderia birmana						X		X		X		X						X	X	
Nylanderia flavipes	X	X	X	X	X	X	X	X	X	X			X							
Nylanderia vividula	X		X	X	X	X	X	X	X	X	X	X	X	X	X	X	X	X	X	X
Odontomachus circulus	X	X	X	X	X	X	X	X	X	X	X									
Odontomachus monticola														X	X	X	X	X	X	
Odontoponera transversa	X	X	X	X	X	X	X	X	X	X	X	X	X	X	X					
Oecophylla smaragdina	X	X	X	X		X														

Paraparatrechina sauteri		X				X	X		X		X	X	X	X	X	X	X	X	X	X
Pheidole cf. *acantha*								X												
Pheidole cf. *quadricuspis*				X																
Pheidole fervens	X	X				X	X	X			X									
Pheidole fortis																X	X	X	X	X
Pheidole gatesi								X							X			X	X	
Pheidole laevicolor													X			X	X	X		
Pheidole ochracea						X	X	X	X	X		X	X	X	X					X
Pheidole parva				X	X				X			X								
Pheidole pieli			X			X	X		X	X	X	X	X	X	X	X	X	X	X	X
Pheidole plagiaria	X	X	X	X	X			X	X											
Pheidole rugithorax											X	X		X	X				X	
Pheidole smythiesii		X	X		X		X	X	X											
Pheidole tjibodana		X	X			X		X	X			X	X			X		X		X
Pheidole tumida			X	X	X	X	X	X		X	X	X			X					
Pheidole vieti	X	X	X	X					X						X					
Pheidole vulgaris		X		X	X	X	X				X			X	X	X	X			X
Pheidole watsoni	X	X	X	X	X	X	X	X	X						X			X		
Plagiolepis alluaudi			X			X	X			X	X	X	X		X			X		
Platythyrea parallela													X							
Polyrhachis armata		X		X			X			X			X	X						
Polyrhachis bicolor															X		X		X	X
Polyrhachis brevicorpa							X		X		X						X		X	
Polyrhachis ceylonensis									X						X			X	X	X
Polyrhachis cf. *clypeata*					X															
Polyrhachis cf. *tibialis*					X					X	X	X			X					
Polyrhachis clypeata				X																
Polyrhachis dentihumera							X	X			X		X	X	X					
Polyrhachis halidayi								X				X				X	X	X	X	X
Polyrhachis illaudata		X		X						X			X				X		X	X
Polyrhachis moesta	X	X		X	X			X	X											
Polyrhachis orbihumera								X												
Polyrhachis phipsoni												X			X				X	
Polyrhachis pubescens								X												
Polyrhachis sp. C		X																		
Polyrhachis thompsoni								X							X		X	X		
Polyrhachis thrinax															X					
Polyrhachis tibialis									X			X								X

Ponera baka		X		X				X												
Ponera cf. *diodonta*												X								
Ponera longlina																	X			X
Ponera menglana		X		X		X				X										
Ponera nangongshana		X																X	X	
Prenolepis emmae												X			X					
Prenolepis magnocula	X	X		X	X		X			X					X					X
Prenolepis naoroji																X			X	
Prenolepis sphingthorax	X	X							X											
Pristomyrmex brevispinosus	X	X							X	X				X	X					
Pristomyrmex hamatus	X			X						X		X	X							
Proceratium cf. *longigaster*															X			X		
Proceratium longigaster		X											X							
Proceratium zhaoi																				X
Pseudolasius silvestrii	X	X		X	X	X	X	X	X	X	X	X	X	X	X					
Pseudoneoponera rufipes							X	X												
Recurvidris recurvispinosa					X	X	X	X			X		X		X	X	X	X		
Rhopalomastix rothneyi											X									
Solenopsis indagatrix	X		X	X	X	X	X			X	X		X							
Strumigenys assamensis				X					X		X	X								
Strumigenys cf. *bryanti*				X	X															
Strumigenys cf. *exhilirina*				X	X			X												
Strumigenys doriae		X																		
Strumigenys mitis	X	X	X		X	X	X		X	X	X					X				
Strumigenys nanzanensis		X	X	X	X		X	X	X	X	X	X	X	X	X	X		X		
Strumigenys nongba												X								X
Strumigenys rallarhina	X	X	X	X	X	X	X	X	X	X										
Strumigenys sauteri			X	X				X	X		X									
Strumigenys strugax	X		X	X	X	X	X	X	X		X	X	X	X	X					
Strumigenys sydorata			X	X			X		X		X									
Strumigenys tritomea				X			X								X					
Tapinoma indicum					X															
Technomyrmex albipes						X	X	X	X		X	X	X	X	X	X	X			
Technomyrmex antennus	X			X	X	X	X		X		X	X	X					X		
Technomyrmex elatior	X			X			X		X											
Technomyrmex kraepelini	X			X			X	X						X	X					
Technomyrmex obscurior	X								X				X	X	X		X			
Technomyrmex pratensis			X								X		X	X	X					

Technomyrmex vitiensis							X							X						
Technomyrmex yamanei						X	X								X					
Temnothorax angulohumerus																				X
Temnothorax cf. *congruus*												X								
Temnothorax cf. *zhejiangensis*													X							
Temnothorax sp. C														X	X					
Tetramorium aptum					X			X	X											
Tetramorium indosinense																X	X		X	
Tetramorium kraepelini					X	X	X	X	X	X		X	X		X					
Tetramorium lanuginosum				X	X													X	X	
Tetramorium laparum						X						X								
Tetramorium melleus										X										
Tetramorium nipponense	X	X	X	X	X		X	X	X	X	X	X	X	X	X	X	X	X	X	X
Tetramorium obtusidens						X														
Tetramorium pacificum															X					
Tetramorium smithi	X	X				X	X	X		X	X		X			X	X	X	X	X
Tetramorium wroughtonii							X						X	X	X					
Tetraponera allaborans									X							X		X		X
Tetraponera attenuata			X	X		X	X		X	X	X	X		X						
Tetraponera furcata							X						X			X	X			
Vollenhovia emeryi	X	X	X	X	X	X	X	X	X	X	X	X	X	X	X					
Vollenhovia lucimandibula							X					X								X
Vombisidris tibeta				X														X		
